# Functional Outcome of Surgically Treated Isolated Coronoid Fractures With Elbow Dislocation in Young and Active Patients

**DOI:** 10.7759/cureus.10883

**Published:** 2020-10-10

**Authors:** Deepak Kumar, Praveen Sodavarapu, Karmesh Kumar, Aman Hooda, Deepak Neradi, Vikas Bachchal

**Affiliations:** 1 Orthopaedics, Postgraduate Institute of Medical Education and Research, Chandigarh, IND

**Keywords:** coronoid fracture, elbow dislocation, lateral collateral ligament

## Abstract

Coronoid fractures are less frequent injuries seen in around one-tenth of patients with elbow dislocation. Any injury to the coronoid process can be associated with elbow instability, in which injury to collateral ligaments co-exists, resulting in a loss of congruency of the elbow joint. However, there is a scarcity of evidence regarding patients' management with elbow dislocation and associated coronoid fractures. So, our aim is to assess the functional outcome of the elbow after operative fixation in patients with any type of coronoid fracture with associated elbow dislocation. A total of six patients with closed coronoid fracture of the elbow, with associated elbow dislocation, without any other associated trauma or previous surgery to the same limb, were included in our study. After closed reduction, patients with an incongruent reduction of the elbow joint were operated. The injured structures were repaired in an inside-out sequence: the coronoid fragment was first reduced by using a lasso-type suture. The larger fragments of the coronoid were fixed with either a screw or a plate when deemed necessary. Then, the lateral collateral ligament was repaired either using a suture anchor or transosseous (No. 2 Arthrex; Naples, Florida) sutures.

After repair, the elbow was examined for stability radiologically using the hanging arm test; a concentric reduction of the elbow in lateral view during this test indicates a stable elbow. All patients showed a good to excellent outcome on the Mayo elbow performance score (MEPS) at the final follow-up (three patients had an excellent score while three had a good score). At the final follow-up, mean elbow flexion was 124º, loss of extension was 10º in only one patient, mean supination was 80º, and mean pronation was 72º. Isolated fractures of the coronoid associated with elbow dislocation require appropriate evaluation and management. Closed reduction and immobilization alone in young and active patients may not be sufficient, especially in patients with incongruent ulnohumeral joint. Surgical fixation of the coronoid fragment and repair of the collateral ligament, whenever indicated, can provide good functional outcomes.

## Introduction

Coronoid fractures are less frequent injuries, which are seen in around one-tenth of patients with elbow dislocation [1-2]. They are commonly affiliated with soft tissue damage around the elbow joint [3]. The coronoid process is a segment of the proximal ulna and stabilizes the elbow joint against forces directed posteriorly [4]. It further provides restraint to varus stress in combination with the lateral collateral ligament (LCL) complex [5]. The coronoid process has three soft tissue insertions: the anterior joint capsule of the elbow, the brachialis muscle, and the medial ulnar collateral ligament (MUCL) [6-7]. MUCL is the principal element in providing elbow stability against valgus stresses [8-9].

Along with the medial and lateral ligament complexes, the coronoid has been recognized as one of the principal components responsible for maintaining elbow stability [10]. Any injury to the coronoid process can be associated with elbow instability, in which injury to these ligaments co-exists, resulting in a loss of congruency of the elbow joint [11-13]. However, there is a scarcity of evidence regarding the management of patients with elbow dislocation and associated coronoid fractures.

So, our aim is to assess the functional outcome of the elbow after operative fixation in patients with any type of coronoid fracture with an associated elbow dislocation, in view of the important role of the coronoid process in maintaining elbow stability.

## Materials and methods

All patients presenting to the tertiary care center between February 2017 and January 2019 with closed elbow dislocation and an associated coronoid fracture were considered for inclusion. After local and neurovascular examination, anteroposterior (AP) and lateral radiographs of the elbow were performed. A total of six patients with a closed coronoid fracture of the elbow with associated elbow dislocation without any other associated trauma or previous surgery to the same limb were included in our study. All demographic data (age, handedness), injury (timeline, mechanism, associated dislocation), and radiographic data (Regan Morrey classification, degree of displacement) were recorded [14]. All six patients underwent closed reduction of the elbow under anesthesia, showed an incongruent reduction of the elbow joint, were unstable on stress testing, and were considered for surgical fixation. Stability was checked in extension and 30 degrees of flexion, and varus and valgus stress were applied.

Operative technique

**Figure 1:**
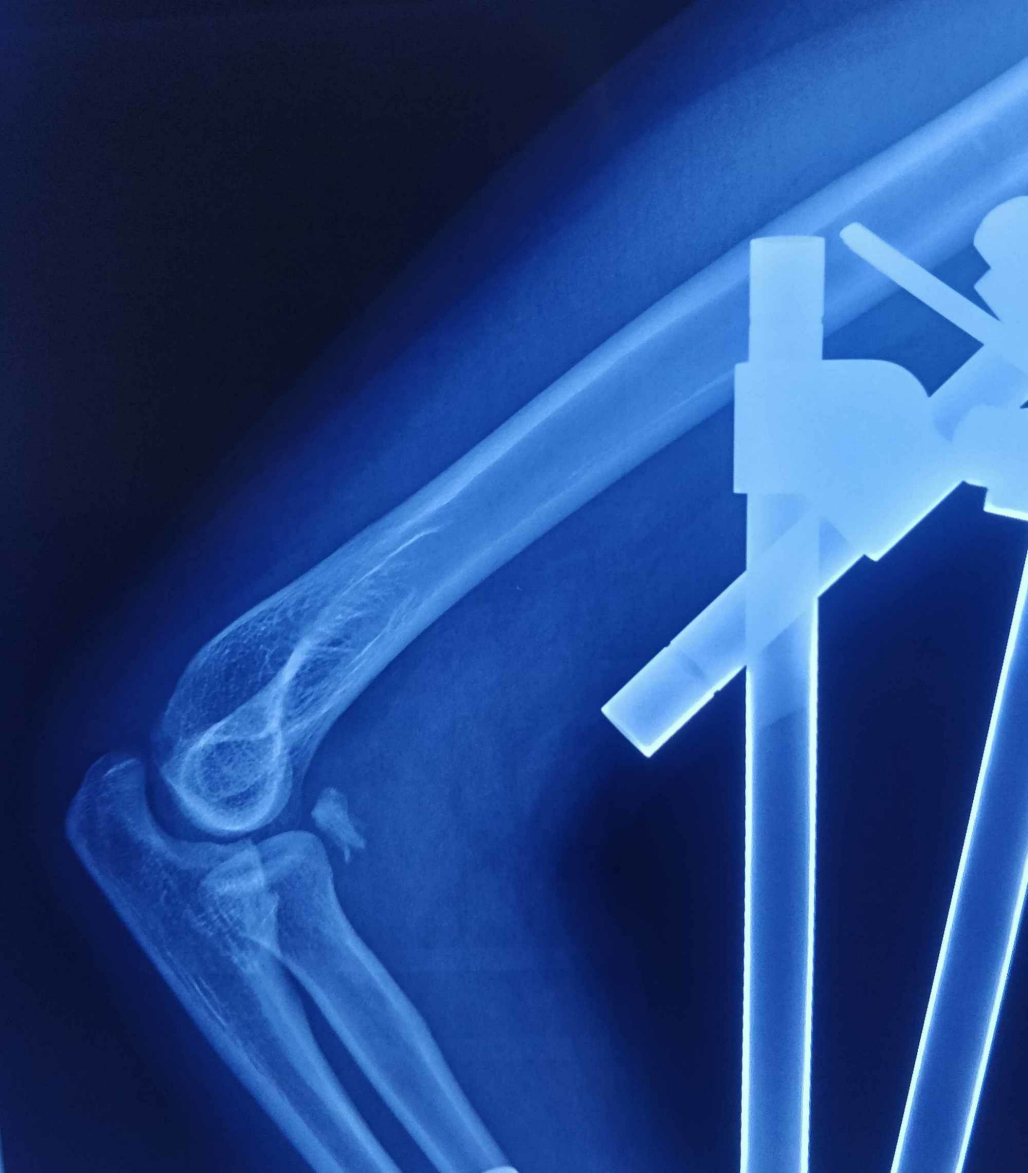
Case 1 - lateral radiograph of the left elbow showing incongruent elbow joint post reduction and application of external fixator

**Figure 2:**
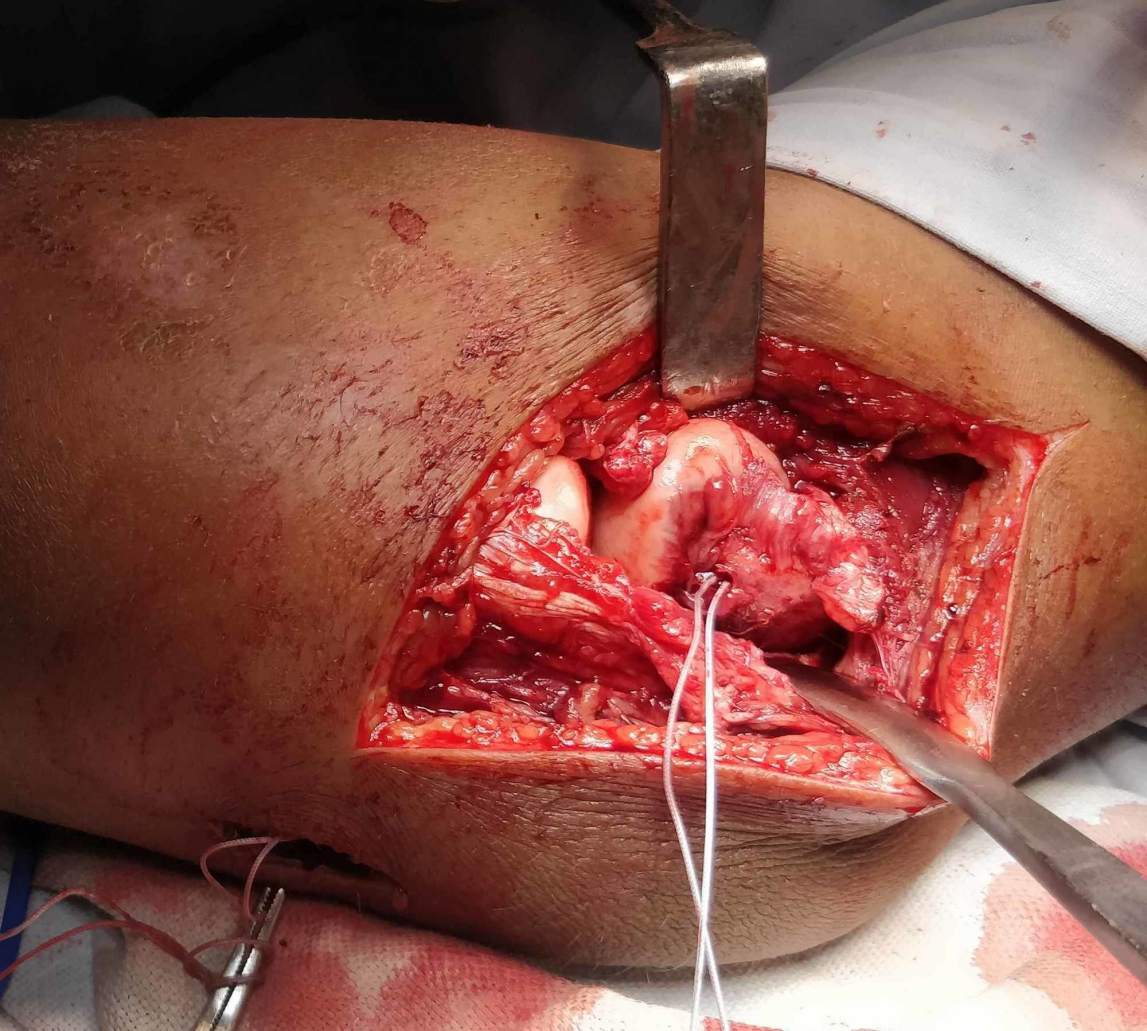
Case 1 - intraoperative photo showing ligamentous repair using suture anchor at the isometric point on the lateral condyle

**Figure 3:**
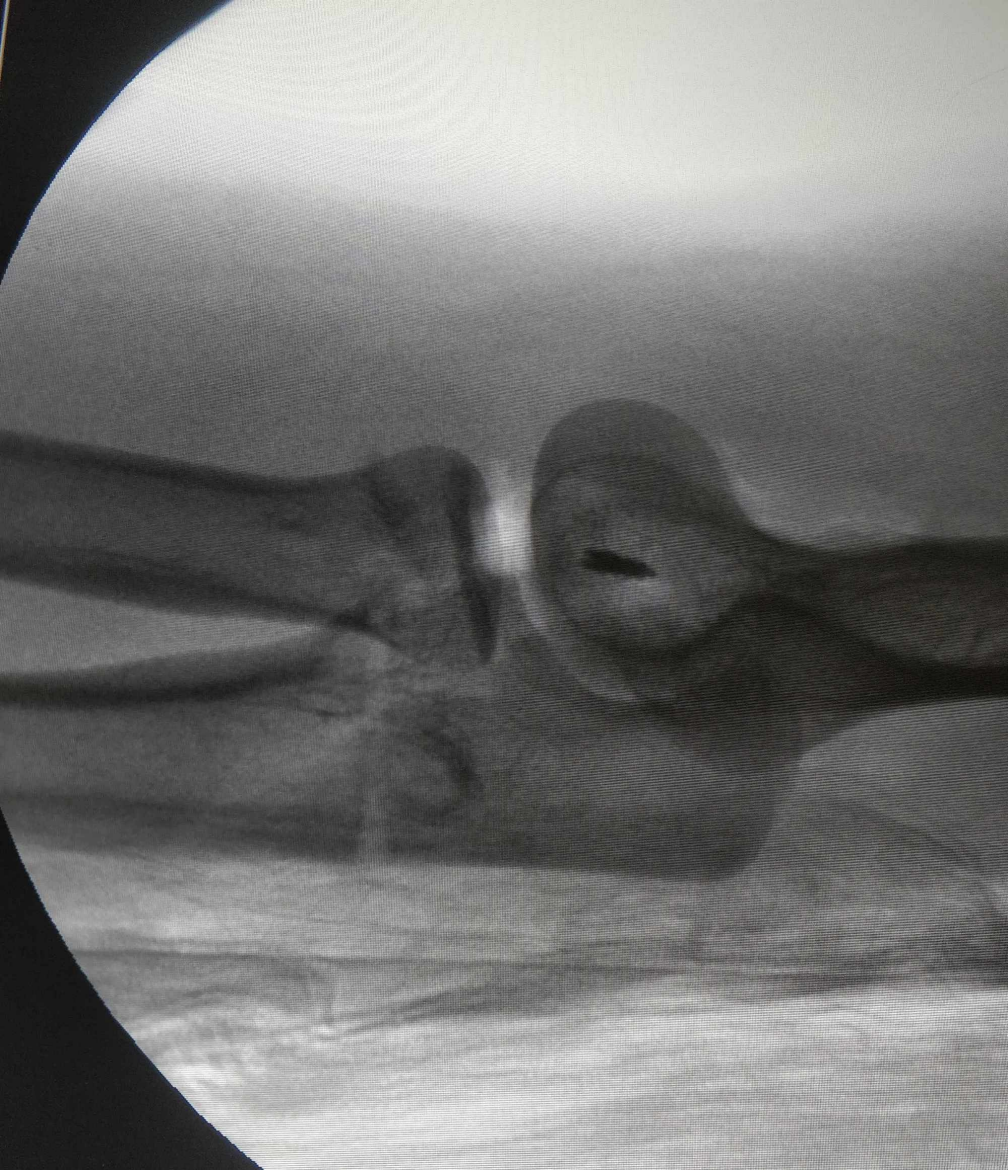
Case 1 - intraoperative fluoroscopic lateral view of elbow showing concentric elbow reduction during the hanging arm test

After closed reduction, patients with an incongruent reduction of elbow joint were operated in the same sitting, except in one patient where an external fixator was applied due to poor soft tissue condition and the operation took place after seven days. All patients were operated in the supine position with elbow positioned over the arm board. The extended Kocher approach was used, utilizing the Kocher interval distally between the extensor carpi ulnaris and anconeus muscles, and proximally between the triceps and mobile wad of Henry in all cases. In addition, two cases required a separate anteromedial approach to gain access to the coronoid fragment. The injured structures were repaired in an inside-out sequence: the coronoid fragment was first reduced by using a lasso-type suture, passed through the anterior capsular attachment, and over the coronoid fragment. Then, a suture passer was used to pass the suture threads along the sides of the coronoid base and through the ulna posteriorly. The suture threads were then tightened to achieve suitable tension and tied over the posterior aspect of the ulna. Larger fragments of coronoid were fixed with either a screw or plate when deemed necessary. Then, the lateral collateral ligament was then repaired either using a suture anchor or using transosseous (No. 2 Arthrex) sutures through the bone tunnels at the isometric point on the lateral condyle. After repair, the elbow was examined for stability radiologically using the hanging arm test, performed with elbow in full extension, and in supination, with sterile towels placed underneath the arm, so that the weight of the hanging limb produces a dislocating force. A c-arm lateral view is checked to ascertain whether the elbow is reduced during this test. A concentric reduction of the elbow in the lateral view during this test indicates a stable elbow (Figures 1-3).

After determining the stability of the elbow, the wound was closed in layers and a posterior splint was given. Patients were given indomethacin 25 mg thrice daily for three weeks prophylactically to prevent heterotopic ossification.

Postoperative protocol

Postoperatively, the elbow was immobilized at 90° of flexion and in a mid-prone position. The splint was retained for 10 days, and then a range of motion (ROM) brace was applied with a 30° extension limit. We encouraged the patient to perform an active range of motion exercises for one month, followed by active assisted exercises for the next two months under a physiotherapist's guidance.

Follow-up and functional evaluation

**Figure 4:**
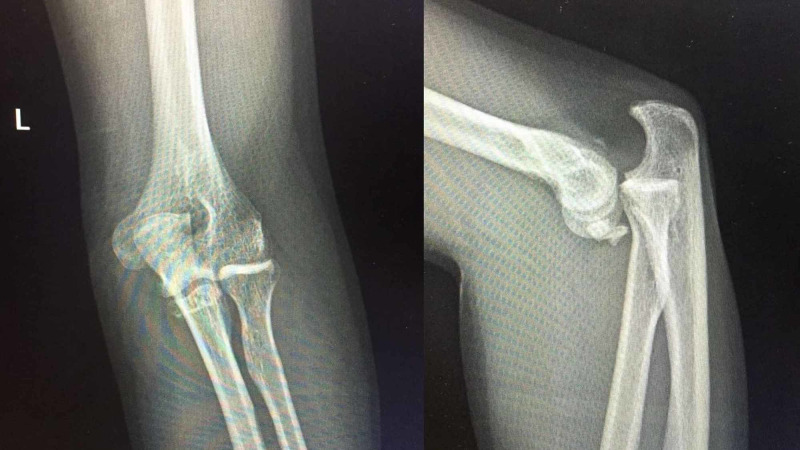
Case 5 - anteroposterior and lateral radiographs of the elbow showing a coronoid fracture with elbow dislocation

**Figure 5:**
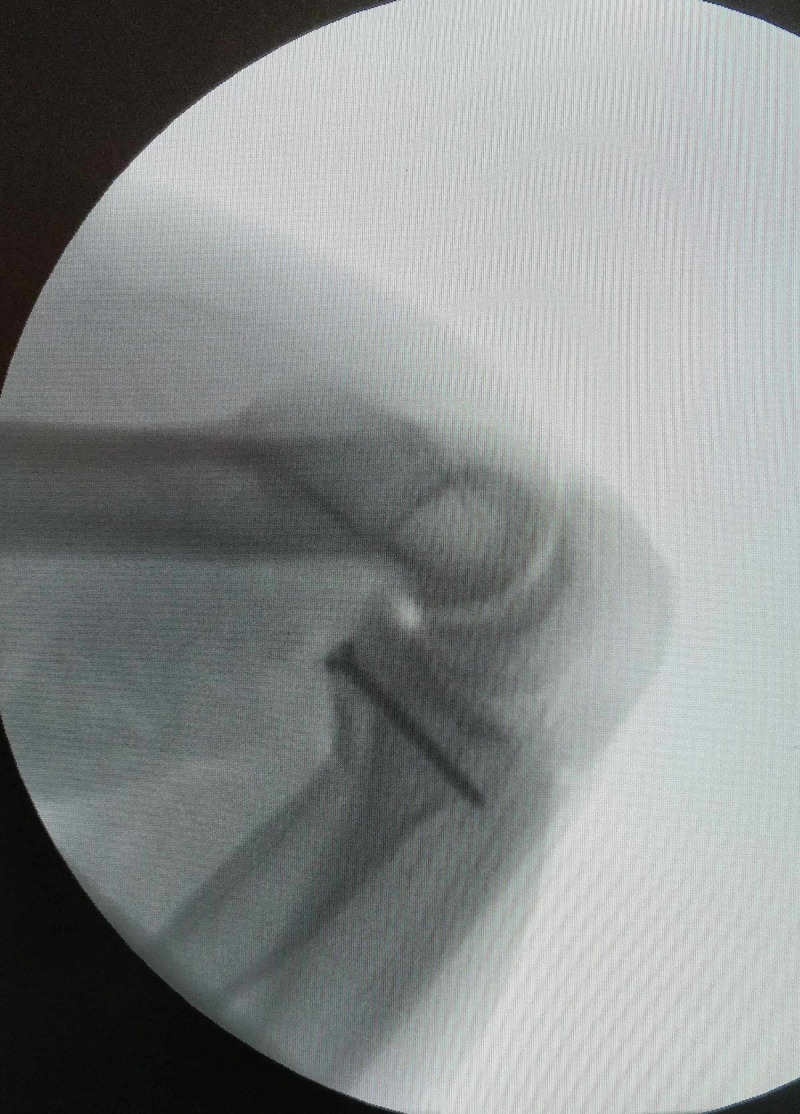
Case 5 - intraoperative c-arm image showing reduced elbow and coronoid fragment stabilized with a screw

**Figure 6:**
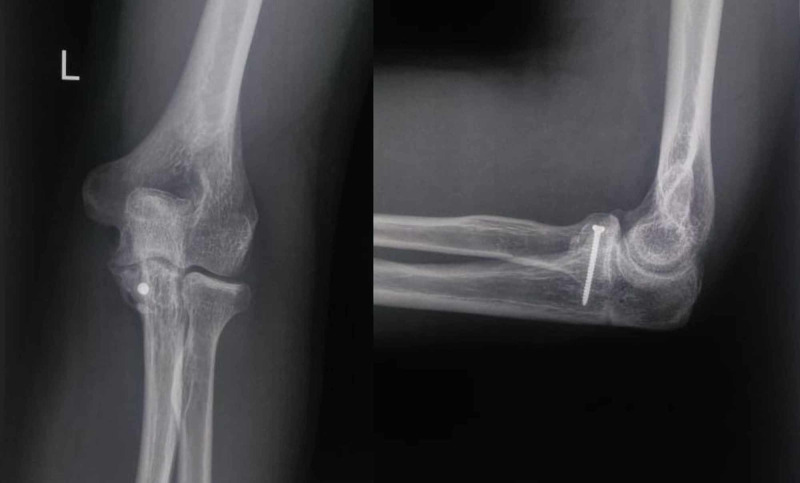
Case 5 - anteroposterior radiograph and lateral radiograph of the elbow at the final follow-up showing reduced elbow with the united coronoid fracture

**Figure 7:**
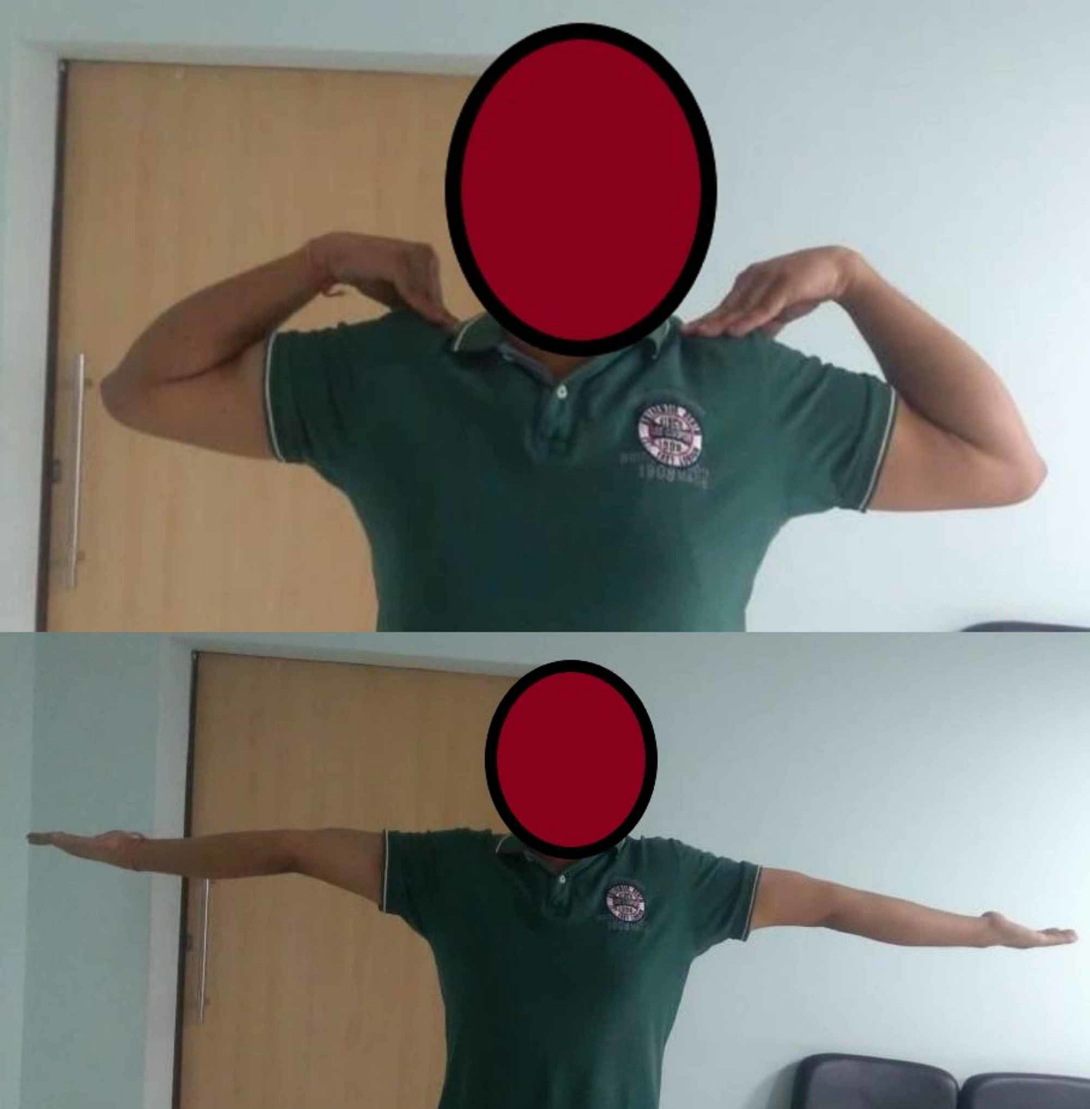
Case 5 - clinical photo at the final follow-up showing complete flexion and extension of the left elbow, comparable to the opposite side

All patients were evaluated clinically and radiographically at two weeks, six weeks, 12 weeks, and at the final follow-up by the same operative surgeon. Patients were clinically assessed by the Mayo elbow performance score (MEPS) [15]. Radiographic evaluation of the elbow by the AP and lateral views was performed at follow-ups to check for joint congruency (Figures 4-7).

## Results

All patients showed a good to excellent outcome on the MEPS at the final follow-up (three patients had an excellent score while three had a good score). At the final follow-up, mean elbow flexion was 124º, loss of extension was 10º in only one patient, mean supination was 80º, and mean pronation was 72º. Radiographs of all patients showed a concentric reduction of the elbow, no signs of post-traumatic arthritis, and heterotrophic ossification. Superficial infection was seen in one patient but resolved with oral antibiotics. All coronoid fractures were united at the final follow-up on radiographs. The demographic and fracture characteristics of all patients are shown in Table 1, and the clinical assessment findings of all patients are shown in Table 2.

**Table 1:** Demographic and fracture characteristics of all patients M - Male, L - left, R - right, ND - non-dominant, D - dominant, RTA - road traffic accident, LCL - lateral collateral ligament, PL -posterolateral, PM - posteromedial

Case	Age/Sex	Side	Mechanism of injury	Regan Morrey type	Elbow dislocation	Associated LCL injury
1.	19/M	L/D	Fall	2	PL	Yes
2.	35/M	R/D	Fall	1	PL	Yes
3.	33/M	L/ND	Fall	3	PM	Yes
4.	23/M	R/D	RTA	1	PL	Yes
5.	26/M	R/D	RTA	2	Posterior	Yes
6.	26/M	R/D	Fall	2	PL	Yes

**Table 2:** Postoperative clinical assessment of all patients MEPS - Mayo elbow performance score, Sup. - Superficial, Postop. - postoperative

Case	Age/Sex	Fixation of coronoid	Approach	Postop. flexion/extension	Postop. supination/pronation	Final follow-up in months	MEPS	Complications
1.	19/M	Suture	Lateral	0º-130º	80º/85º	23	95	Nil
2.	35/M	Suture	Lateral	0º-130º	90º/80º	20	95	Nil
3.	33/M	Plate	Lateral + Anteromedial	0º-135º	90º/80º	18	100	Nil
4.	23/M	Suture	Lateral	10º-110º	70º/60º	15	85	Sup. infection
5.	26/M	Screw + Suture	Lateral+ Anterior	0º-125º	80º/70º	15	85	Nil
6.	26/M	Suture	Lateral	0º-120º	70º/60º	13	85	Nil

## Discussion

The coronoid process is one of the critical structures for maintaining elbow stability and forms an anterior buttress to prevent the elbow's posterior dislocation. It also acts as an important constraint against axial, varus, and posterolateral forces [7]. The coronoid fracture is closely related to the capsule of the elbow joint, as well as the medial and lateral collateral ligament complexes, and hence an isolated coronoid fracture seldom occurs in isolation. They are less frequent injuries, which are seen in around one-tenth of patients with elbow dislocation [16]. The role of the coronoid process in elbow stability against axial, posterolateral rotatory, or varus loads was studied in previous biomechanical and cadaver studies, and structures that have attached to the coronoid process play an important role in elbow stability [17-19]. According to Morrey et al., 50% of the height of the coronoid process is essential to make sure of elbow stability in the sagittal plane [20]. Most coronoid fractures occur in patients who are involved in a high level of physical activity. Fixation of such fractures can be essential, especially in associated elbow dislocation, to achieve a congruent ulnohumeral articulation, maintain elbow stability, and reduce the chances of arthritis. A computed tomography (CT) scan is useful to assess the fracture configuration and intra-articular involvement. It gives more details than radiographs regarding the articular compression, displacement, and comminution of the fracture. Also, the presence of any intra-articular fragment is revealed, which may hinder a congruent reduction of the elbow joint.

In general, a fracture with greater than 50% of ulnar involvement can be managed surgically, to achieve an early range of motion. Fractures less than 50% of height have to be evaluated for ligament injury and elbow instability. Associated elbow dislocation and the presence of incongruent elbow reduction indicate a requirement for surgical treatment with necessary ligament repair. For larger fragments (Regan Morrey types 2 and 3), screw or mini-fragment plates can be used and for the smaller fragments (Regan Morrey types 1 and 2), Herbert screws, or repair using the lasso suture technique can be done.

Only a few studies are available in the literature with isolated coronoid fractures with or without elbow dislocation. Foruria et al. studied 38 consecutive patients sustaining acute isolated coronoid fractures in which 28 were treated nonsurgically and obtained good results in most of the patients [21]. He suggested that an isolated coronoid fracture with a congruent elbow joint, an adequate sublime tubercle, and a fractured coronoid height of less than 50% can be treated nonoperatively. However, only eight patients were associated with elbow dislocation. Also, four patients required surgery in the follow-up, and it is not clear how many patients with associated dislocation required any subsequent surgery. Another study by Kekatpure et al. evaluated the incidence of combined osteochondral and ligamentous injuries by magnetic resonance imaging (MRI) in 24 patients with isolated coronoid fracture without elbow dislocation and detected an LCL injury in all the patients [22]. Any injury to the coronoid, small or large, with associated elbow dislocation, has a higher likelihood of ligamentous injury and may induce an inherent instability of varying degrees, which can lead to post-traumatic arthritis. Hence, a better outcome can be expected in such patients with surgical intervention, which provides stability and allows early joint mobilization.

In our study, all patients were young and active, and the dominant limb was involved in the majority of them. The type of elbow dislocation was predominantly posterolateral, and the mode of injury was due to a fall on an outstretched hand. Post-reduction, all elbows were unstable and the joint was incongruent and, therefore, required surgical intervention. We classified the fracture based on the Regan and Morrey classification according to which two patients with type 1, three patients with type 2, and one patient with type 3 coronoid fractures were seen. As we couldn't obtain humeral subtraction views of the ulna on CT scan, the fractures were not classified according to O'Driscoll. Fixation of the coronoid fractures was done by a mini fragment plate in one patient, by screws in two patients, and by the suture lasso technique in the rest of the patients. All our cases had an LCL injury, which can be attributed to the mechanism of injury. The lateral collateral ligament was repaired by FiberWire (Arthrex) in all cases to achieve lateral stability. No operative neurovascular and postoperative complications were noted except a superficial infection in one case but did not require any further operative procedure. We initiated active ROM exercises from day one as tolerated by patients under adequate analgesia, in order to avoid a stiff elbow [1].

All patients showed good to excellent functional outcomes on the MEPS. Although our final follow-up was limited, with a maximum follow-up of 23 months, no signs of post-traumatic arthritis were seen in radiographs at the final follow-up. At the final follow-up, all patients showed a good functional range of motion and no residual elbow instability.

## Conclusions

Any injury with an isolated coronoid fracture should not be overlooked, as they can appear naïve on radiographs and can be associated with the ligamentous injury. Isolated fractures of the coronoid associated with elbow dislocation require appropriate evaluation and management. Closed reduction and immobilization alone in young and active patients may not be sufficient, especially in patients with an incongruent ulnohumeral joint. Surgical fixation of the coronoid fragment and the collateral ligament repair, whenever indicated, can provide good functional outcomes.
